# Factors associated with moderate or severe left atrioventricular valve regurgitation within 30 days of repair of complete atrioventricular septal defect

**DOI:** 10.5935/1678-9741.20150036

**Published:** 2015

**Authors:** Marcelo Felipe Kozak, Ana Carolina Leiroz Ferreira Botelho Maisano Kozak, Carlos Henrique De Marchi, Sirio Hassem Sobrinho Junior, Ulisses Alexandre Croti, Airton Camacho Moscardini

**Affiliations:** 1 Department of Pediatrics and Pediatric Surgery, Hospital de Base, São José do Rio Preto Medical School, São José do Rio Preto, SP, Brazil.; 2 Department of Cardiology, Hospital de Base, São José do Rio Preto Medical School, São José do Rio Preto, SP, Brazil.

**Keywords:** Endocardial Cushion Defects, Mitral Valve Insufficiency, Postoperative Period

## Abstract

**Introduction:**

Left atrioventricular valve regurgitation is the most concerning residual lesion after surgical correction of atrioventricular septal defects.

**Objective:**

To determine factors associated with moderate or severe left atrioventricular valve regurgitation within 30 days of surgical repair of complete atrioventricular septal defect.

**Methods:**

We assessed the results of 53 consecutive patients 3 years-old and younger presenting with complete atrioventricular septal defect that were operated on at our practice between 2002 and 2010. The following variables were considered: age, weight, absence of Down syndrome, grade of preoperative atrioventricular valve regurgitation, abnormalities on the left atrioventricular valve and the use of annuloplasty. Median age was 6.7 months; median weight was 5.3 Kg; 86.8% had Down syndrome. At the time of preoperative evaluation, there were 26 cases with moderate or severe left atrioventricular valve regurgitation (49.1%). Abnormalities on the left atrioventricular valve were found in 11.3%; annuloplasty was performed in 34% of the patients.

**Results:**

At the time of postoperative evaluation, there were 21 cases with moderate or severe left atrioventricular valve regurgitation (39.6%). After performing a multivariate analysis, the only significant factor associated with moderate or severe left atrioventricular valve regurgitation was the absence of Down syndrome (*P*=0.03).

**Conclusion:**

Absence of Down syndrome was associated with moderate or severe postoperative left atrioventricular valve regurgitation after surgical repair of complete atrioventricular septal defect at our practice.

## Abstract (pt)

**Introdução:**

A insuficiência da valva atrioventricular esquerda é a lesão residual mais preocupante após o tratamento cirúrgico do defeito de septo atrioventricular.

**Objetivo:**

Determinar fatores associados à insuficiência da valva atrioventricular esquerda de grau moderado ou importante nos primeiros 30 dias após correção de defeito de defeito de septo atrioventricular total.

**Métodos:**

Avaliamos os resultados em 53 pacientes consecutivos menores de 3 anos com defeito de septo atrioventricular total, operados em nosso serviço entre 2002 e 2010. Avaliamos as seguintes variáveis: idade, peso, ausência de síndrome de Down, grau de insuficiência da valva atrioventricular esquerda antes da correção, anormalidades na valva atrioventricular e uso de anuloplastia. A mediana da idade foi de 6,7 meses e a do peso de 5,3 Kg; 86,8% tinham síndrome de Down. Antes da operação, 26 apresentavam insuficiência da valva atrioventricular esquerda pelo menos moderada (49,1%). Anormalidades na valva atroventricular foram encontradas em 11,3% dos casos; anuloplastia foi realizada em 34% dos pacientes.

**Resultados:**

Após a correção, houve 21 casos com insuficiência moderada ou grave da valva atrioventricular esquerda (39,6%). Após realização de análise multivariada, o único fator associado com esses graus de insuficiência foi a ausência da síndrome de Down (*P*=0,03).

**Conclusão:**

Ausência de síndrome de Down esteve associada com insuficiência moderada ou grave da valva atrioventricular esquerda após correção cirúrgica de defeito de septo atrioventricular total em nosso serviço.

**Table:** 

**Abbreviations, acronyms & symbols**	
AV	Atrioventricular
AVSD	Atrioventricular septal defect
LAVVR	Left atrioventricular valve regurgitation

## INTRODUCTION

In North America and Europe, between 9% and 17% of patients with complete atrioventricular septal defect (AVSD) are discharged home with significant residual left atrioventricular valve regurgitation (LAVVR) after definitive surgical repair, even with intraoperative monitoring by transesophageal echocardiography^[1,2]^. Aside from the obvious risk of reoperation that it implies, the presence of LAVVR with hemodynamic compromise in this period may increase length of hospital stay, morbidity and mortality, and it may also increase costs^[1-6]^.

In order to improve outcomes, a clear outline of the predisposing factors leading to residual LAVVR after surgical repair is mandatory. The most common risk factors associated with reoperation are: abnormalities on the atrioventricular (AV) valve, non-closure of the zone of apposition of the AV valve, absence of Down syndrome, low weight, preoperative AVVR, age lower than 3 months at time of repair and a more acute angle of the AV valve^[1,4,7-10]^. However, there are few studies aimed to detect factors associated with significant immediate postoperative LAVVR^[5]^. The purpose of our study was to assess whether some of the risk factors for reoperation previously published in the literature would be associated with an at least moderate LAVVR within 30 days of surgical repair of complete AVSD at our practice.

## METHODS

This study was approved by the ethics committee of our institution (protocol CEP 3802/2010), a tertiary-care hospital with a division of pediatric cardiology and cardiovascular surgery in Brazil, which waived the need for patient consent. The medical records of all patients 14 years old and younger who had undergone repair of complete AVSD at our practice between March 2002 and April 2010 were reviewed. Patients with any right ventricle obstruction, and those who had a previous pulmonary banding were excluded.

The reports of the transthoracic echocardiograms performed before and after operation were reviewed. These exams were performed by one of two physicians using commercially available machines, HDI 5000CV (ATL Ultrasound), Envisor-C and HD11 (Philips Ultrasound, Bothell, WA, USA), with 3 to 8 MHz probes. For further analysis, there were considered the exam before surgery and the exam closer to the 30^th^ postoperative day, while still being within 1 month of the repair. Transesophageal echocardiographic probe was not available at our institution during the period in which the patients were operated on.

Pre- and postoperative AVVR were subjectively divided into 4 grades based on the appearance of the color Doppler jets in relation to the surrounding chambers. I: absent or trivial; II: mild; III: moderate; IV: severe^[11]^. The categorizations were based just on official written summaries of the exams. Images stored on tapes or in digital media were not assessed. The mechanisms of AVVR were described in a few cases, therefore these information were not used for analysis.

The following risk factors were assessed: age, weight, absence of Down syndrome, grade of preoperative AVVR, abnormalities on the AV valve morphology and the need for the use of annuloplasty. Abnormalities on the AV valve morphology were subjectively described by the surgeon.

### Statistical Analysis

Continuous variables were expressed as median, and comparisons were made using the two-sided Mann-Whitney test. Cathegorical variables were expressed using frequency distribution and percentages, and comparisons were made using the Fisher exact test. Univariate odds ratios and their 95% confidence intervals (95% CI) were estimated for variables found to have a statistically significant (*P*≤0.05) relationship with moderate or severe postoperative LAVVR. These variables were included in the multivariate analysis when they reached a *P*-value≤0.2. A P-value of 0.05 or less was considered significant. All statistical analyses were conducted using the software StatsDirect, version 2.7.2. 2008 (Cheshire, UK).

### Patient Population

We included 53 patients (37 girls and 16 boys): 46 with Down syndrome (86.8%). Age at the time of repair ranged from 2.7 months to 3 years (median 6.7 months); 37.7% of the patients were 6 months-old or younger, and 83% were 1 year-old or younger. Weight varied between 2.9 and 13 Kg (mean 5.3) (Table 1). At the time of preoperative evaluation, there were 4 cases with grade I AVVR (7.5%), 23 with grade II (43.4%), 16 with grade III (30.2%), and 10 with grade IV (18.9%). Patients with Down syndrome had lower grades of preoperative AVVR than those without Down syndrome, but it was not statistically significant (*P*=0.2). Six cases (11.3%) of abnormalities on the AV valve morphology were found: small left AV valve orifice (3), hypoplastic left mural leaflet (1), accessory cleft (1), and grossly malformed valve (1). Among the 46 patients with Down syndrome, 5 (10.9%) had abnormalities on the AV valve, whereas 1 out of the 7 patients (14.3%) without Down syndrome had these abnormalities (*P*=0.99).

**Table 1:** Characteristics of the 53 patients enrolled in the study.

Characteristic	N (%)
Age at the time of repair in months (median)	6.7 (2.7-35.6)
Female	37 (69.8)
Weight in Kg (median)	5.3 (2.9-13)
Down syndrome	46 (86.8)
Grade 1 AVVR	4 (7.5)
Grade 2 AVVR	23 (43.4)
Grade 3 AVVR	16 (30.2)
Grade 4 AVVR	10 (18.9)
AV valve abnormalities	6 (11.3)

AVVR=atrioventricular valve regurgitation; AV=atrioventricular

### Operative and postoperative management

Surgery using a median sternotomy was performed in all patients. Continuous extracorporeal circulation by ascending aortic and bicaval cannulation with deep hypothermia (rectal temperature 22ºC) was used in 2 patients, while moderate hypothermia (rectal temperature 25-28ºC) was used in 51 patients. Cardiopulmonary bypass time varied between 66 and 200 minutes (median 105 min), and the aortic cross-clamp time varied between 42 and 180 minutes (median 78.5 min). Antegrade cold crystalloid cardioplegia was used at 20-minute intervals for myocardial preservation.

The two-patch technique with preserved bovine pericardium was used in 50 patients (94.3%); the single-patch modified technique was used in the other 3 patients, in whom the surgeon considered the ventricular septal defect too small.

The zone of apposition or cleft was completely closed in 49 patients (92.4%) and tt was partially closed in 1 patient. It was left open in another 3 patients: in 2 patients the valve annulus was very small, and in another patient the posterior annuloplasty had reduced the diameter of the valve orifice, what made the cleft closure more difficult to be done. Posterior annuloplasty was performed in 18 patients (34%) who presented annular dilation and valve leaking after repair, based on surgical saline testing.

## RESULTS

The postoperative time on a mechanical ventilator ranged from 4 to 1699.3 hours (median 19.9 hours) and the time of inotropic support varied between 24 and 1146 hours (median 81 hours). The postoperative length of hospital stay ranged from 1 to 149 days (mean 11.5 days). There were 4 deaths (7.1%): one within the first 24 hours due to cardiogenic shock; another due to a complete AV block and cardiogenic shock at the 12^nd^ postoperative day before pacemaker implantation; one due to sepsis at the 24^th^ postoperative day and another at the 25^th^ postoperative day due to multi-systemic organ failure. The postoperative LAVVR grades of these patients who died were II, I, III and IV respectively (their preoperative AVVR grades were respectively III, III, III and II).

The echocardiograms considered for analysis were performed between day 1 and day 29 after repair (mean 12.1±8.5 days). At the time of postoperative evaluation, there were 5 cases with grade I LAVVR (9.4%), 27 with grade II (50.9%), 18 with grade III (34%), and 3 with grade IV (5.7%). The difference between pre- and postoperative grades of AVVR was marginally significant (*P*=0.06). There was a partial or complete improvement of AVVR or maintenance of a trivial or mild AVVR in 35 patients (66%). A one-grade worsening was found in 12 patients (22.6%).

Among the 4 cases in which the cleft was left partially or completely open, there was no difference between pre- and postoperative LAVVR (*P*>0.99). However, among those in which the cleft was closed, this difference was significant (*P*=0.05). Regarding those who had undergone annuloplasty, the difference between pre- and postoperative LAVVR was not significant (*P*=0.4), and among those who had not undergone annuloplasty, this difference was only marginally significant (*P*=0.08).

Other findings on postoperative echocardiograms included the following: 10 cases of right AVVR (18.9%), 7 cases of residual small ventricular septal defects (13.2%), 2 cases of residual small atrial septal defects (3.8%) and 1 case of left AV valve stenosis (1.9%).

According to the univariate analysis, absence of Down syndrome was the only factor associated with moderate or severe LAVVR after surgical repair (*P*=0.01). Presence of mild or more severe preoperative AVVR and presence of AV valve abnormalities were only marginally significant (Table 2). Under multivariate analysis, only absence of Down syndrome continued to be associated with moderate or severe postoperative LAVVR (*P*=0.03) (Table 3).

**Table 2:** Univariate analysis of preoperative and intraoperative factors related to postoperative left atrioventricular valve regurgitation grade moderate or severe.

Factor	LAVVR ≤ II	LAVVR ≥ III		Univariate	
	(n=32)	(n=21)	OR	IC (95%)	*P*
Age in months (median)	7.7	6.1			0.63
Weight in Kg (median)	5.4	5			0.93
Down syndrome	31 (96.9%)	15 (71.4%)	0.08	0.002-0.791	0.01
Preoperative AVVR ≥ II	28 (87.5%)	21 (100%)			0.14
Annuloplasty	11 (34.4%)	7 (33.3%)			> 0.99
AV valve abnormality	2 (6.2%)	4 (19%)			0.20

AV=atrioventricular; AVVR=atrioventricular valve regurgitation; CI=confidence interval; LAVVR=left atrioventricular valve regurgitation; OR=odds ratio

**Table 3:** Multivariate analysis of preoperative and intraoperative factors related to postoperative left atrioventricular valve regurgitation grade moderate or severe.

Factor	PO LAVVR ≤ II	PO LAVVR ≥ III		Multivariate	
	n=32	n=21	OR	CI 95%	*P*
Non-Down syndrome	1 (3.1%)	15 (28.6%)	0.08	0.009-0.774	0.03
Preoperative AVVR ≥ II	28 (87.5%)	21 (100%)			0.99
AV valve abnormality	2 (6.2%)	4 (19%)			0.21

AV=atrioventricular; AVVR=atrioventricular valve regurgitation; CI=confidence interval; PO LAVVR=postoperative left atrioventricular valve regurgitation; OR=odds ratio

## DISCUSSION

Even in the modern era, with the use of new medical diagnostic tools as routine intraoperative transesophageal echocardiography or 3D-chocardiography, and with the use of different surgical techniques, the risk of late reoperation for LAVVR continues high after repair of AVSD^[1,7,9,12-15]^. Although rare, early reoperations for LAVVR also happen^[16-18]^. This is true for both complete and incomplete forms of AVSD, despite different physiology and different ages at repair. What both forms of AVSD have in common are the typical anatomical landmarks of AVSD (common AV junction, a common 5-leaflet AV valve, distinct papillary muscle displacement and a narrow and elongated left ventricle outflow tract), as well as a high prevalence of individuals with Down syndrome^[19]^. Therefore, the clue to understanding this frequent complication may be related more to these two aspects than to another factor such as age at repair, weight at repair, or AV valve malformation.

Statements such as "In patients with Down-syndrome valve tissue is more abundant and allows for an easier reconstruction" are often seen^[1]^. It suggests that patients without Down syndrome would have a higher risk of worse surgical outcomes regarding LAVVR, as found by us and some other authors^[9,12,13]^. In the study by Ferrín et al.^[20]^, it was found that patients with Down syndrome indeed have more tissue in the AV valve, but also a lower prevalence of malformation of the AV valve, what not necessarily meant a better outcome. In the study by Kanani et al.^[21]^, in which the anatomy of the subvalvar apparatus of normal hearts was compared to that of hearts with AVSD, the structural and geometric disarray of the tendinous cords of the hearts with AVSD was clearly visible, along with its possible role on the mechanisms of valve regurgitation. However, there was no mention if patients with Down syndrome were included in the study. In the study by Desai et al.^[16]^, comparing Down and non-Down patients, there were differences neither with respect to grade of preoperative LAVVR, nor with respect to the prevalence of dysplastic AV valves. Studies addressed to explain this very common finding, like histopathological comparisons of the valve and subvalvar apparatus of patients with and without Down syndrome, must be done or, maybe, the surgical approach to patients without Down syndrome should be revisited.

Significant preoperative AVVR eventually leads to left ventricular dilation, changing the cordal axis, influencing the mechanism of valve closure, what could be responsible for persistent postoperative LAVVR^[21]^. In our study, the presence of an at least mild preoperative AVVR was not associated with moderate or severe postoperative LAVVR. It is important to mention that none of the patients enrolled in our study presented left ventricular dysfunction at the moment of postoperative assessment, what could skew the results. Also, in a more recent work by Bharucha et al.^[10]^, the grade of preoperative AVVR didn't influence the results. With the use of three-dimensional (3D) echocardiography, they found that a more acute angle of the components of the common AV valve against the plane of the common AV junction would be a predictor of postoperative valve function. Unfortunately, 3D-echo was not available at our institution when the study was performed, therefore this risk factor could not be addressed.

The results of our study could not prove any relationship between the presence of AV valve malformation and moderate or severe postoperative LAVVR. Some studies found that AV valve malformation is associated with reoperation or valve replacement^[7,9]^. However, like in our study some cases of AV valve malformation have been subjectively diagnosed^[7]^. Ando & Takahashi^[22]^ found a weak correlation between preoperative echocardiographic findings and the surgeon's judgment in regard to the diagnosis of these malformations. Furthermore, there is little consistency between the findings of two- and three-dimensional (2D and 3D) echocardiography in respect to the analyses of AV valve abnormalities. In the study by Takahashi et al.^[23]^, for instance, the correlation between the findings of both methods was lower than 46% in the examination of the mural leaflet and in the examination of the commissural abnormalities of the left AV valve leaflets. Three-dimensional echocardiogram was more accurate and more reliable. In another study by Takahashi et al.^[24]^, the overall sensitivity for the assessment of left AV valves using 2D (transthoracic and transesophageal) and 3D-echocardiography was less than 60%, although the specificity and accuracy of 3D-echocardiography were superior, providing complementary information with this relatively new method.

With respect to the approach of the zone of apposition, some studies have reported that not closing it would be a risk factor of reoperation, and it has therefore been adopted at our institution^[14,25]^. However, in 4 cases in this study (7.5%) the cleft was not completely closed due to the risk of stenosis. In these cases, there was no alteration in the functional status of the valve, differently of those who had this procedure performed, who clearly had some benefit from this approach. The rate of annuloplasty in this study (34%) was similar to that reported by Suzuki et al.^[7]^ (30%), and much higher than those reported by Dragulescu et al.^[15]^ (4.7%) and by Stellin et al.^[2]^ (2.5%). These different rates show that there is no common sense on this item. Padala et al.^[26]^ showed, *in vitro*, the importance of the annular dilation for the AVVR grade: they concluded that performing only the cleft closure is not enough to avoid AVVR, and that there would have a real benefit in shortening the annulus size. Such benefit was not found in the present study.

### Study Limitations

It was a retrospective study, and it was therefore subject to limitations in terms of how correctly the information in the medical records was well filled. The study is limited by the relatively low number of patients and by the lack of immediate postoperative data provided by an intraoperative transesophageal echocardiogram. The postoperative echocardiograms were performed anytime during the first 30 postoperative days, rather than a consistent postoperative interval. It is important since the same patient behaves differently depending on his/her hemodynamical situation, usually more stable the later the evaluation is done. Statements made by the surgeon were merely subjective and the mechanisms of LAVVR were not available for a better understanding of our results, clearly not as good as expected.

## CONCLUSION

Absence of Down syndrome was associated with moderate or severe postoperative LAVVR in patients with complete AVSD operated on at our practice. We suggest that these patients must have a detailed preoperative assessment of their AVV morphology and whenever possible an intraoperative transesophageal echocardiography in order to immediately assess the surgical result and minimize the effect of the genetic background on their surgical outcomes.

**Table:** 

**Authors’ roles & responsibilities**	
MFK	Analysis and/or interpretation of data; statistical analysis; final approval of the manuscript; study design; writing of the manuscript or critical review of its content
ACLFBMK	Analysis and/or interpretation of data; final approval of the manuscript; writing of the manuscript or critical review of its content
CHM	Analysis and/or interpretation of data; final approval of the manuscript; writing of the manuscript or critical review of its content
SHSJ	Analysis and/or interpretation of data; final approval of the manuscript; writing of the manuscript or critical review of its content
UAC	Analysis and/or interpretation of data; final approval of the manuscript; implementation of projects and/or experiments; writing of the manuscript or critical review of its content
ACM	Analysis and/or interpretation of data; final approval of the manuscript; study design; writing of the manuscript or criti-cal review of its content
